# The Impact of Virgin and Aged Microstructured Plastics on Proteins: The Case of Hemoglobin Adsorption and Oxygenation

**DOI:** 10.3390/ijms25137047

**Published:** 2024-06-27

**Authors:** Florent Saudrais, Marion Schvartz, Jean-Philippe Renault, Jorge Vieira, Stéphanie Devineau, Jocelyne Leroy, Olivier Taché, Yves Boulard, Serge Pin

**Affiliations:** 1 NIMBE, CNRS, CEA, Université Paris-Saclay, 91191 Gif-sur-Yvette, France; florent.saudrais@cea.fr (F.S.);; 2 Unité de Biologie Fonctionnelle et Adaptative, CNRS, Université Paris Cité, 75013 Paris, France; 3 Institute for Integrative Biology of the Cell (I2BC), CNRS, CEA, Université Paris-Saclay, 91198 Gif-sur-Yvette, France

**Keywords:** hemoglobin, microstructured plastics, hemoglobin adsorption, hemoglobin activity, oxygenation

## Abstract

Plastic particles, particularly micro- and nanoparticles, are emerging pollutants due to the ever-growing amount of plastics produced across a wide variety of sectors. When plastic particles enter a biological medium, they become surrounded by a corona, giving them their biological identity and determining their interactions in the living environment and their biological effects. Here, we studied the interactions of microstructured plastics with hemoglobin (Hb). Virgin polyethylene microparticles (PEMPs) and polypropylene microparticles (PPMPs) as well as heat- or irradiation-aged microparticles (ag-PEMPs and ag-PPMPs) were used to quantify Hb adsorption. Polypropylene filters (PP-filters) were used to measure the oxygenation of adsorbed Hb. Microstructured plastics were characterized using optical microscopy, SAXS, ATR-FTIR, XPS, and Raman spectroscopy. Adsorption isotherms showed that the Hb corona thickness is larger on PPMPs than on PEMPs and Hb has a higher affinity for PPMPs than for PEMPs. Hb had a lower affinity for ag-PEMPs and ag-PPMPs, but they can be adsorbed in larger amounts. The presence of partial charges on the plastic surface and the oxidation rate of microplastics may explain these differences. Tonometry experiments using an original method, the diffuse reflection of light, showed that adsorbed Hb on PP-filters retains its cooperativity, but its affinity for O_2_ decreases significantly.

## 1. Introduction

Plastics are a family of synthetic organic polymers (polyethylene, polypropylene, polystyrene, polyurethane, polyvinyl chloride, etc.) [1]. Without additives, plastic materials are not viable [2]. Additives are added during the production process to modify physical or chemical properties of plastics and increase their performance and long-term stability (plasticizers, heat or light stabilizers, antioxidants, flame retardants, colorants, etc.) [3]. Due to the variety of properties that can be obtained using different polymers and additives, plastic materials are produced in very large quantities and used in a wide range of industrial sectors, such as packaging, building and construction, textiles, transportation, etc. [4].

Worldwide, it has been estimated that between 8300 and 9200 Mt of plastics had been produced by 2017 [5]. Nevertheless, since 2000 over 400 Mt has been produced each year, equaling the amount that was produced in the previous 50 years [4,5]. The longevity of plastics is one of their main characteristics but also a cause of problems. They are not biodegradable and can accumulate overtime. Plastics are therefore a major source of pollution and, by 2015, have led to an estimated 6300 Mt of plastic waste [4,5], with only 9–10% of all plastics being recycled, 12–15% being incinerated, and 75–79% ending up in dumps, landfills, and waterways [4,5].

These uncontrolled plastics are subject to chemical and physical environmental factors, such as UV radiation and mechanical forces in the aquatic environment, which cause their aging. In general, the main effects include the oxidation of polymers, the breaking of chemical bonds, the modification of their crystalline structure, and the adsorption of polar and apolar compounds [6,7,8]. The fragmentation of macroscopic plastic debris results in the formation of microscopic and nanoscopic particles, called micro- and nanoplastics (MNPs) [9].

MNP contamination is ubiquitous worldwide [10,11], and like the amount of plastic waste, the amount of these plastic particles has been increasing for several decades [12,13,14]. MNPs, once in water, soil, and the atmosphere, can be ingested or inhaled by animals and humans [15,16,17]. MNPs can be transferred from soil to animals and humans via plants or absorbed by marine organisms, such as plankton and algae, and then introduced into the food chain [18,19,20,21,22,23]. Humans can similarly be directly exposed to and ingest MNPs by eating food contaminated by plastic packaging or through drinking water [24,25,26,27]. As a result, it has been estimated that up to 4.7 × 10^3^ microplastics are ingested each year per person via drinking water and up to 3.0 × 10^7^ by inhalation [28]. Moreover, microplastics can accumulate in the case of chronic exposure [29,30].

The human exposure to more or less aged plastic particles by ingestion and inhalation (as well as via skin contact) raises questions about their impact on the human body and harmful risks to health [31,32,33,34,35,36]. Moreover, this potential toxicity may depend on the toxic additives contained in MNPs [37,38,39] or other pollutants (metals, organic pollutants) sorbed on MNPs [39,40,41]. A large variety of health effects have been observed after MNP exposure depending on the nature and the size of particles. These observations involve the system, organ, and cellular levels as well as bodily fluids [31,32,33,34,35,36]. MNPs can translocate through lung, gastrointestinal, and skin epithelia into the blood capillary system and then into the circulatory system [36,42,43,44,45]. A double-shot pyrolysis gas chromatography–mass spectrometry study on a set of 22 adult donors showed that the mean concentration of all plastic particles is 1.6 μg.mL^−1^ in the blood [46]. Studies have shown that MNPs can adsorb on red blood cells via non-covalent interactions [47,48] and destabilize their lipid membrane by mechanical stretching [49]. In addition, plasma proteins have a strong affinity for plastic particles [50,51,52,53].

Because plastics are not chemically reactive and due to the large specific surface area of small particles (e.g., in the micron and submicron range), their biological effects result from interactions at the molecular level at the interface with biomolecules. Like all nanomaterials, once in biological media, plastic particles are covered by a layer of molecules mostly composed of proteins. This protein corona can very efficiently stabilize MNPs and determine their uptake and their biological outcome [54,55,56,57]. Protein adsorption on organic or inorganic nanoparticles has long been known to cause changes in protein structure and activity [58,59]. Although studies on the adsorption of proteins on MNPs and adsorption-induced protein modifications have been constantly increasing, published results are still sparse. In particular, there is little information on the physicochemical parameters of adsorption and changes in the activity of adsorbed proteins. For example, studies have recently shown that hemoglobin may interact with plastic particles (polyethylene microplastics and polystyrene nanoplastics) via hydrophobic or van der Waals forces or hydrogen bonding and cause changes to the hemoglobin structure [60,61,62]. These studies suggest that MNPs interact with hemoglobin, producing structural alterations that may consequently affect the functional characteristics of the protein.

Here, we sought to investigate the mechanism of hemoglobin adsorption on MNPs and to analyze the effects of adsorption on hemoglobin activity. Hemoglobin is a very well-known protein, and its structural, functional, and adsorption properties have been extensively characterized. Its oxygen-binding function is particularly amenable to studying protein activity after adsorption, the gaseous ligand preventing any interference with plastics during the experimental measurements [63,64,65]. Polyethylene (PE) and polypropylene (PP) microparticles (PEMPs and PPMPs) were chosen because PE and PP are the most produced plastics and are persistent; therefore, they dominate plastic pollution [4]. Moreover, the PEMPs and PPMPs used in this study come from the same batches as in our previous study [57]. To assess the effects of plastic aging on interactions with hemoglobin, PEMPs and PPMPs were aged in controlled and reproducible manners: irradiation or heat oxidation, respectively. The aged particles (ag-PEMPs and ag-PPMPs) can thus contain ketone, hydroxyl, carboxylic, or ester groups [66,67]. For tonometry experiments, sample oxygenation was carried out on polypropylene microstructured filters (PP-filters) using an unusual technique, the diffuse reflection of light. Our results suggest that hemoglobin is adsorbed on MNPs, causing changes in activity whose origin can be traced to structural alterations of the protein.

## 2. Results and Discussion

### 2.1. Characterization of Microstructured Plastics

#### 2.1.1. Characterization of Aged Microparticles

To understand the interactions of hemoglobin with microstructured plastics, it is important to characterize the plastic samples used. Here, we used the same batches of virgin PE and PP microparticles whose features were thoroughly described in our previous study [57]. In addition to optical microscopy, which provided information on the morphology and the size of plastic microparticles, we used small-angle X-ray scattering (SAXS) to obtain information on their surface structures at the nanometric scale, X-ray photoelectron spectroscopy (XPS) for details on their surface compositions, and Fourier-transform infrared (FTIR) spectra and Raman spectra to specify their chemical compositions [57]. These details are summarized Table 1. For comparison, Table 2 gives the characteristics obtained on the aged PE and PP microparticles using the same techniques and the same methods.

Microscopy experiments performed on the DISCO beamline of the Synchrotron SOLEIL (Saint Aubin, France) show that these virgin microplastics have an irregular rounded shape [57]. However, PP microparticles are larger than PE microparticles, with median Feret diameters of 10.0 μm and 4.2 μm, respectively (Table 1). The sizes of both types of microplastic are polydisperse. SAXS-measured specific surface areas are 2.5 m^2^.g^−1^ for PEMPs and 1.1 m^2^.g^−1^ for PPMPs (Table 1). The comparison of these surface areas with the calculated values (1.5 m^2^.g^−1^ and 0.7 m^2^.g^−1^) for perfect PEMP and PPMP spheres using the measured radii from Table 1 suggests that the virgin microparticle surface is relatively smooth, with low roughness [57]. PEMPs and PPMPs exhibit ATR-FTIR spectra known for PE and PP. No additives or impurities were detected in any of the PE and PP microparticles using FTIR [57]. Using XPS, only carbon is found on the PPMP surface, and oxygen, representing 0.9% of the signal, is identified in addition to carbon on the PEMP surface (Table 1). This result indicates limited oxidation of the surface of virgin PE microparticles [57]. The Raman spectrum of PEMPs corresponds to those of PE found in the literature. Confocal Raman imaging identified three different spectral components sharing the same vibrational bands but different intensity ratios, and these components correspond to different degrees of PE crystallinity. On the other hand, the Raman imaging of PPMPs shows that only one component is observed, whose Raman spectrum is consistent with spectra reported in the literature [57].

**Figure 1:**
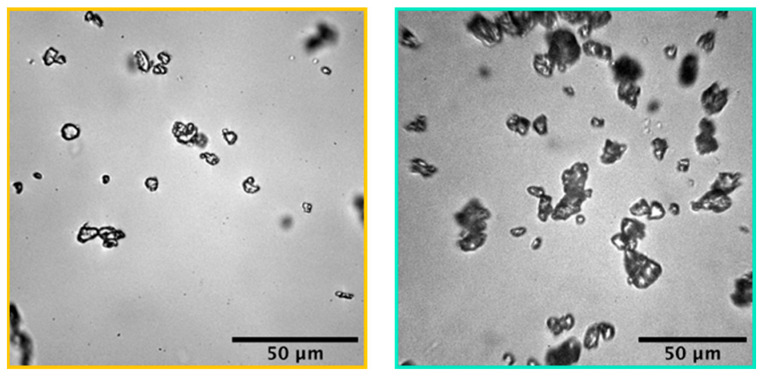
Microscopic visible images of aged PE microparticles (**left**) and aged PP microparticles (**right**).

The microscopy experiments showed that the aged PE and PP microparticles always had different morphologies and polydispersed sizes (Figure 1). However, ag-PEMPs were larger than their virgin counterparts, with a median Feret diameter increasing from 4.2 to 5.8 μm after aging (Table 1 and Table 2). This increase in size suggests a possible agglomeration or aggregation of PE particles during aging. On the other hand, aging had no effect on the size of ag-PPMPs with a diameter of 9.9 µm for 10.0 µm before aging (Table 1 and Table 2).

The SAXS spectra of PE microparticles before and after aging were similar (Figure S1). The specific surface area of ag-PEMPs was determined, and the values before and after aging were similar, 2.5 and 2.4 m^2^.g^−1^, respectively (Table 1 and Table 2). The surface structure of PEMPs did not seem to have been significantly modified during aging. By contrast, the SAXS spectra of PP microparticles were very different before and after aging (Figure S1). In particular, the ag-PPMP spectrum did not display the signal around 0.1 Å^−1^ present in the PPMP spectrum, suggesting that the internal structure of PP microparticles was homogenized during heat-aging. However, the values of the specific surface area of PP microparticles before and after aging were similar (1.1 and 1.2 m^2^.g^−1^, respectively (Table 1 and Table 2)).

After irradiation (2 MGy) or heat treatment (85 °C for two months), the ATR-FTIR spectra of ag-PEMPs and ag-PPMPs showed a peak at 1715 cm^−1^ [68], characteristic of carbonyl groups. The carbonyl index (CI), defined as the ratio between the integrated absorbance band of the (C=O) peak from 1850 to 1650 cm^−1^ and that of the methylene (CH_2_) scissoring peak from 1500 to 1420 cm^−1^ [69], was calculated to compare the plastics’ oxidation rate. CI increased from 0.00 ± 0.02 to 0.73 ± 0.04 after the irradiation of PEMPs and from 0.02 ± 0.02 to 1.15 ± 0.04 after the heat treatment of PPMPs (Table 1 and Table 2). Using the ATR-FTIR spectrum of 3-octanone and its calculated CI as a reference, we estimated that the aged microparticles contained between 3 and 4.8% oxygen atoms.

As in the case of virgin PEMPs [57], carbon (around 285 eV) and oxygen (around 533 eV) were found using XPS on the surface of ag-PEMPs (Figure S2). Two oxygen contributions were detected; the first one at 534 eV may correspond to ester groups (-O-C=O) and the second at 532 eV to carbonyl groups (-C=O) [70]. The oxygen signal of ag-PEMPs represented 2.6% of the signal, highlighting an increase in the oxidation of PEMPs, which contained only 0.9% oxygen atoms (Table 1 and Table 2). Similarly to ag-PEMPs, carbon (around 285 eV), and oxygen (around 233 eV) were found on the surface of ag-PPMPs (Figure S3); however, only one oxygen contribution was detected. Whereas PPMPs do not contain oxygen atoms, ag-PPMPs were well oxidized and contained 2.6% oxygen atoms (Table 1 and Table 2). XPS measurements provided a better evaluation of the amount of oxygen atoms in the aged microparticles than the FTIR experiments.

There were no significant differences between the Raman spectra of the ag-PEMPs (Figure S4) and that of virgin PEMPs in [57]. The confocal Raman microscopy of ag-PEMPs made it possible to identify the three different spectral components observed with PEMPs. Similarly, there were no differences between the Raman spectra of ag-PPMPs (Figure S5) and PPMPs [57], and only one spectral component was observed in both the virgin and aged PP microparticles.

#### 2.1.2. Characterization of Polypropylene Filters

Like the (PE or PP, virgin or aged) microparticles, PP microstructured filters were systematically characterized using the same techniques. The results are given in Table 1 for comparison with PPMPs. However, although fibers could be observed using optical microscopy, their diameter of about 2 µm seems to correspond to the resolution limit of the microscope. For this reason, the images of filters were not exploitable and will therefore not be shown here.

From the SAXS measurements on PP-filters, we calculated $Iq^{4}$, where I corresponds to the absolute scale scattering intensity (in cm^−1^ units) and q to the scattering wave vector (in cm^−1^ units). This value was then plotted as a function of q in Å^−1^ units for the x-axis (Figure S6). Based on the plateau that appears for q values lower than 2.5 × 10^−2^ Å^−1^, we calculated the specific surface area of filters using Equation (1) [71]:

(1) $$\begin{array}{c} \sigma_{m}=\frac{\lim\left(Iq^{4}\right)}{2\pi \rho_{m}{\left(∆\rho b\right)}^{2}}\;,\; \end{array}$$

where $\sigma_{m}$ is the specific surface area (in m^2^.g^−1^), lim($Iq^{4}$) is the plateau intensity level, ∆ρb is the interface scattering length (8.72 × 10^10^ cm^−2^ for PP), and $\rho_{m}$ is the mass density (0.9 g.cm^−3^ for PP). We calculated that the specific surface area of the PP-filters is 0.7 m^2^.g^−1^ (Table 1). This value revealed the high specific surface area of filters, where a piece of 10 mm by 15 mm was weighed at 22.5 mg, corresponding to a theoretical specific surface area of 0.0067 m^2^.g^−1^ if the filter surface was not microstructured.

The ATR-FTIR spectrum of PP-filters (Figure S7 and Table S1) presented no significant differences from the spectrum measured on virgin PPMPs [57] and was coherent with pure PP. The filters did not contain additives or impurities that were detectable by FTIR, and their carbonyl index was zero (Table 1). The XPS spectrum of PP-filters confirmed these results (Figure S8). The signal corresponding to the carbon was a single peak consistent with pure PP, and there was no measurable oxygen on the surface of the filters, confirming that the filters were not oxidized.

The Raman spectrum measured for the PP-filters (Figure S9 and Table S2) showed no significant differences from that of PPMPs [57], with all the main peaks corresponding to the PP signal and no unexpected peaks that could indicate the presence of other species. Unlike virgin PPMPs, it was impossible to conduct a full Raman imaging measurement on a PP-filter because the prolonged use of the laser caused it to start burning. We did, however, measure more than 15 individual Raman spectra in different parts of the sample without seeing any significant change in the relative intensity of the different peaks. Therefore, it is likely that there is only one phase in the PP-filter.

### 2.2. Hemoglobin Adsorption on Microparticles

#### 2.2.1. Hemoglobin Adsorption on Virgin Microparticles

The red color of oxyHb offers a means of the direct observation of Hb adsorption. To quantitatively evaluate the plastic–Hb interactions, we used the adsorption isotherm. The Hb adsorption on plastic microparticles was quantified using the depletion method. After the contact of the virgin microplastics with Hb and sample centrifugation to remove the plastic microparticles via creaming, the non-adsorbed Hb concentrations were determined using visible absorption. Then, the amount of adsorbed Hb was calculated from the initial amount of Hb and the amount of non-adsorbed Hb. The adsorption isotherm corresponds to the amount of adsorbed Hb as a function of the free Hb concentration and can be fitted using the Langmuir model, which assumes that adsorption is reversible and limited to a monolayer of non-interacting molecules (see Equation (2)). Two adsorption parameters were thus determined: the maximum amount of adsorbed Hb (m_∞_ in mg.m^−2^) and the adsorption constants (K_ads_ in L.mg^−1^), which are proportional to the Hb affinity for a plastic microparticle.

**Figure 2:**
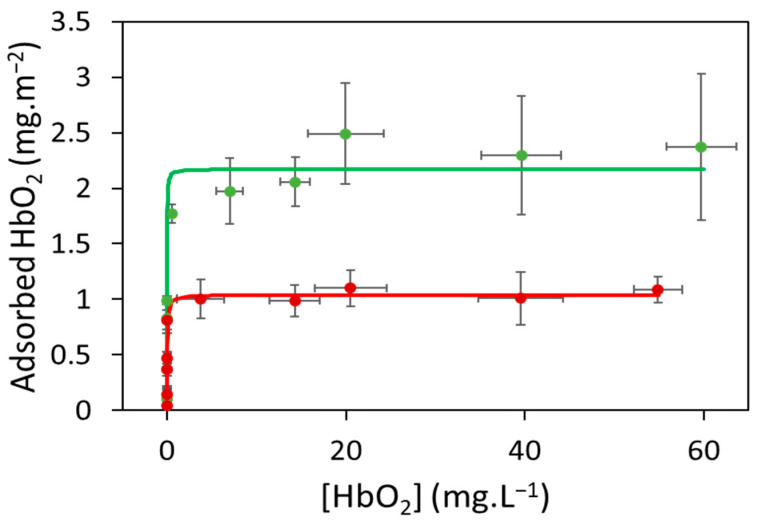
The adsorption isotherms of oxyhemoglobin on virgin polyethylene microparticles (PEMPs) (red circles) and on polypropylene microparticles (PPMPs) (green circles) in 0.1 mol.L^−1^ phosphate buffer pH 7.0. Experimental data (circles) and error bars correspond to the average and standard deviation of three biological replicates. Data were fitted using the Langmuir adsorption model (solid lines).

The adsorption isotherms of oxyHb on PEMPs and PPMPs in 0.1 mol.L^−1^ phosphate buffer pH 7.0 are given in Figure 2. For both plastic microparticles, the results demonstrated that at high Hb concentrations, a hemoglobin layer or hemoglobin corona forms on PEMPs and PPMPs. However, the maximum amount of adsorbed Hb on PPMPs was 2.2 ± 0.2 mg.m^−2^ and higher than that on PEMPs, 1.0 ± 0.1 mg.m^−2^ (Table 3). These m_∞_ values indicated that the thickness of the Hb corona is larger on PPMPs than on PEMPs. In addition, the adsorption constant (Table 3) also appeared significantly higher for PPMPs than for PEMPs (92 ± 8 L.mg^−1^ and 26 ± 3 L.mg^−1^, respectively), indicating that Hb has a higher affinity for PPMPs. Although Hb had a lower affinity for PEMPs, its adsorption kinetics were slower than that on PPMPs, allowing time for adsorbed Hb to cover a larger area before the adsorption of additional molecules and thus leading to a lower amount of Hb adsorbed on PEMPs. In other words, the slower adsorption kinetics of Hb may allow for a better organization of the molecules on the particle surface.

The surface compositions of PEMPs and PPMPs were different (Table 1). PPMPs contain only carbon atoms, whereas PEMPs also contain oxidation sites (0.9% oxygen atoms). The differences in adsorption isotherms (amount of adsorbed Hb and/or the difference in Hb affinity) may be due (at least in part) to the difference in the charge of the PEMP and PPMP surfaces. Furthermore, compared to PE, PP has an additional methyl group, which influences surface properties for adsorption.

To compare the maximum amount of adsorbed Hb to the amount that can be adsorbed on a plastic microparticle to form a monolayer of Hb, we assumed that the microparticles are spherical and that the Hb molecule is a sphere, whose radius is equal to its radius of gyration. With the molecular weight of Hb, we can calculate the m_∞_ value corresponding to a saturated monolayer. Although the number of Hb molecules that can be adsorbed in a monolayer depends on the microparticle radius, the m_∞_ value is independent of this radius because it is relative to the microparticle surface area. The expected m_∞_ value for a saturated monolayer of Hb on the surface of PEMPs and PPMPs is of the order of 2.5 mg.m^−2^. Compared with the values obtained experimentally (1.0 ± 0.1 mg.m^−2^ for PEMPs and 2.2 ± 0.2 mg.m^−2^ for PPMPs, Table 3), our results suggest that the Hb molecules form a saturated monolayer on the PPMP surface.

In a pioneering study [72], Hb adsorption on three polymer films was detected using electrophoresis, and the quantitative measurements of Hb adsorption on these surfaces were obtained based on radioactivity. The values of Hb adsorbed on the three polymers (polyethylmethacrylate, polyhydroxyethylmethacrylate, and PE) and our values of adsorbed Hb on PEMPs and PPMPs are of the same order (0.7–2.8 mg.m^−2^).

Because previous studies focused on serum albumin adsorption on the surface of polymer materials [73], we compared our results found on Hb with those obtained on bovine serum albumin (BSA) with the same batches of PEMPs and PPMPs in the same buffer [57]. The m_∞_ values obtained with Hb match those with BSA. The value measured with PPMPs was higher than that with PEMPs (2.6 ± 0.2 and 1.5 ± 0.1 mg.m^−1^, respectively), indicating that the BSA corona is thicker on PPMPs than on PEMPs as observed for the Hb corona. Moreover, BSA molecules form a saturated layer on the PPMP surface [57]. Even if the K_ads_ value measured on BSA is higher with PPMPs than with PEMPs (8 × 10^2^ ± 3 × 10^2^ and 5 × 10^2^ ± 1 × 10^2^ L.mg^−1^, respectively), both values are an order of magnitude larger than those measured with Hb.

The porcine oxyHb tetramer is structurally similar to that of mammalian Hb [74], and its isoelectric point is around 7.2, whereas that of BSA is 4.8 [75]. In the phosphate buffer pH 7.0 used for these experiments, BSA is therefore more strongly charged than Hb. The differences in adsorption isotherms may be due (at least in part) to the difference in protein charges. Interestingly, these different adsorption behaviors associated with different protein charges have already been observed for porcine oxyHb adsorption on silica nanoparticles [65]. In that study, the adsorption isotherms were measured as a function of pH, and the amount of adsorbed Hb revealed a maximum adsorption from pH 6.2 to pH 7.3 and then a decrease from pH 7.3 to pH 8.5 until no Hb adsorption. The affinity constant K_ads_ of oxyHb adsorption on SIO_2_ nanoparticles decreases as the pH increases from pH 7.0. The adsorption decreases from the isoelectric point of oxyHb. Therefore, we suggest that the protein adsorption on virgin PEMPs (with 0.9% oxygen atoms on their surface) and PPMPs also depends (at least in part) on the value of the global charge of the protein.

#### 2.2.2. Hemoglobin Adsorption on Aged Microparticles

**Figure 3:**
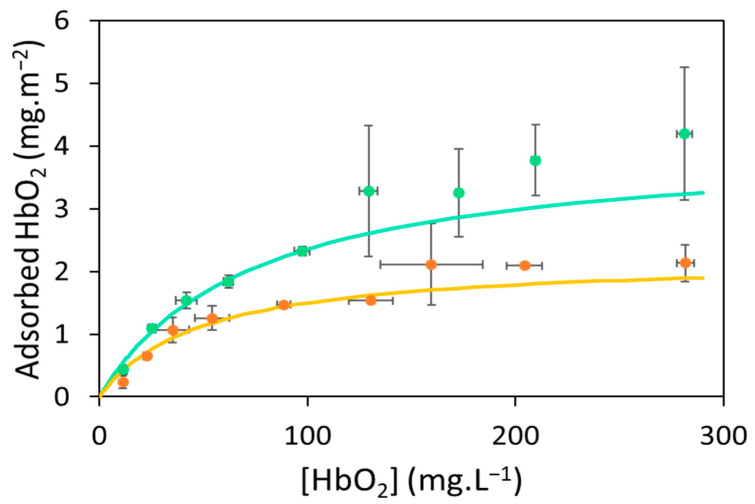
The adsorption isotherms of oxyhemoglobin on aged polyethylene (PE) microparticles (ag-PEMPs) (orange circles) and polyethylene (PP) microparticles (ag-PPMPs) (teal circles) in 0.1 mol.L^−1^ phosphate buffer pH 7.0. Experimental data (circles) and error bars correspond to the average and standard deviation of three biological replicates. Data were fitted using the Langmuir adsorption model (solid lines).

After the contact of the aged microplastics with Hb, the cream is less stable than with virgin microplastics, and the microparticles return more easily in solution. After sample centrifugation to remove plastic microparticles via creaming, it was very difficult to skim them from the surface of the solutions. Therefore, we determined the non-adsorbed Hb concentrations using diffuse reflection spectroscopy (see Section 3.4 and Section 3.5). The adsorption isotherms of oxyHb on ag-PEMPs and ag-PPMPs in 0.1 mol.L^−1^ phosphate buffer pH 7.0 are presented in Figure 3.

Compared with the isotherms measured on the virgin microparticles, the amount of adsorbed Hb increased slowly with Hb concentration, and a plateau was reached for higher Hb concentrations. The isotherms were fitted using the Langmuir model to compare the adsorption parameters with those obtained for PEMPs and PPMPs. All parameters are compiled in Table 3. The K_ads_ and m_∞_ values clearly indicate that Hb had a lower affinity for ag-PEMPs and ag-PPMPs than their virgin counterparts but was adsorbed in larger amounts. More specifically, the maximum amount of adsorbed Hb increased by a factor of 2 (from 1.0 ± 0.1 to 2.2 ± 0.2 mg.m^−2^ for ag-PEMPs and from 2.2 ± 0.2 to 4.1 ± 0.2 mg.m^−2^ for ag-PPMP). However, the variation of the adsorption constant was particularly high and decreased by a factor of 1000 for ag-PEMPs (from 26 ± 3 to 0.021 ± 0.005 L.mg^−1^) and even a factor of 10,000 for ag-PPMPs (from 92 ± 8 to 0.014 ± 0.002 L.mg^−1^).

The main differences between PE and PP microparticles before and after aging (Table 1 and Table 2) were the significant presence of oxygen atoms (2.6%) on their surfaces and the values of carbonyl index (0.73 ± 0.04 for ag-PEMPs and 1.15 ± 0.04 for ag-PPMPs), highlighting the oxidation of aged plastics. Polar carbonyl groups cause the presence of partial charges on microparticle surfaces, which may modify the aged plastic–Hb interactions. However, the variations of K_ads_ and m_∞_ values were unexpected (Table 3). Hb showed lower affinity for aged plastics; the kinetics of its adsorption may thus be slower than those on virgin plastics, and lower amounts of Hb would be adsorbed on aged plastics (see Section 2.2.1). Nevertheless, surface–protein interactions are influenced by the surface properties, including surface energy, polarity, charge, and morphology [76]. In addition, proteins exhibit a less organized structure upon adsorption on a hydrophobic surface than on a hydrophilic surface [77]. Therefore, even though the maximum amount of adsorbed Hb increased, we suggest that oxidation sites on the surface of the aged microparticles limit the overlap of the layer of Hb molecules and/or induce Hb conformational changes.

Recent studies on the interactions of hemoglobin and albumin with microplastics (MPs) and nanoplastics (NPs) have used various spectroscopic analytical techniques to explore the interaction between plastics and proteins. The formation of a hemoglobin corona has been examined on PE MPs and polystyrene NPs [60,61,62]. Remarkably, the interactions of Hb with plastics depend in part on the available surface area [62]. Fluorescence, circular dichroism, and FITR approaches confirm that plastic–Hb interactions cause conformational alterations in Hb, particularly secondary structural modifications. The structural differences may affect Hb functional properties [62]. Using the same spectroscopic techniques, interactions of albumin on PE or polyvinyl chloride MPs and on polystyrene NPs also show albumin corona formation and secondary and tertiary structural changes in protein that may eventually induce function variations [50,51,52,53]. Analyses of interactions of catalase with polyvinyl chloride MPs [78], ovalbumin with polystyrene and polyethylene terephthalate MPs [79], and amylase, peroxidase, or urease with polystyrene NPs [80,81,82,83] lead to the same conclusions on protein corona formation, protein structure, and conformational alterations and the effect on protein activity.

Although there are many studies on the conformational effects of protein adsorption on MNPs, only a few studies have really investigated the variations in protein activity associated with these structural changes. These studies report that the enzyme activity of amylase [80,81] and peroxidase [82] are inhibited by polystyrene NPs, whereas polyvinyl chloride MPs do not affect the catalase activity [78]. Regarding the two model proteins—hemoglobin and albumin—only the catalase-like activity of hemoglobin [62] and the esterase-like activity of albumin [52] have been used to investigate the effect of protein adsorption on protein functionality. The catalase-like activity of hemoglobin is promoted in the presence of polystyrene NPs [62], but they reduce the esterase activity of albumin [52]. Here, in contrast, the oxygen-carrier function was first experimentally studied before and after Hb adsorption on a microstructured plastic (see Section 2.3).

The complex relationship between structural and functional modifications of proteins adsorbed on MNPs has already been observed with protein adsorption on silica NPs. The secondary structural and conformational modifications depend on the nature, size, shape, and surface chemistry of NPs, and the protein activity can be decreased, increased, or can remain constant after adsorption [65].

### 2.3. Hemoglobin Oxygenation on Polypropylene Filters

The suspensions obtained after the contact of oxygenated hemoglobin (oxyHb) with microplastics were unstable and therefore unsuitable for measuring oxygenation curves. Nevertheless, after the contact of oxyHb with plastics (72 h on rotating wheel), the samples were centrifuged to remove the PEMPs or PPMPs via creaming and keep only the non-adsorbed Hb solutions (see Section 3.4 and Section 3.5). We carried out classical tonometry experiments using absorption spectroscopy on these non-adsorbed Hb solutions (Hb after contact with PEMPs and Hb after contact with PPMPs).

To measure the oxygenation curves of hemoglobin adsorbed on microplastics, we chose polypropylene microstructured filters (PP-filters), whose characteristics were very similar to those of PEMPs and PPMPs (Table 1). The contact was made in the same way by replacing the PEMPs or PPMPs with pieces of PP-filters. After the contact of oxyHb with PP-filters (72 h on rotating wheel), we kept the non-adsorbed Hb solution (Hb after contact with PP-filter) for classical tonometry experiments, and the PP-filters themselves were blotted with absorbent paper to remove the solution left on the surface and retain only the adsorbed Hb (see Section 3.5). Each PP-filter was placed in a tonometer with 100 µL of 0.1 mol.L^−1^ phosphate buffer pH 7.4 to prevent the PP-filter from drying out (see Section 3.5). We performed novel tonometry experiments using diffuse reflection spectroscopy on these adsorbed Hb samples (Hb adsorbed on PP-filter).

**Figure 4:**
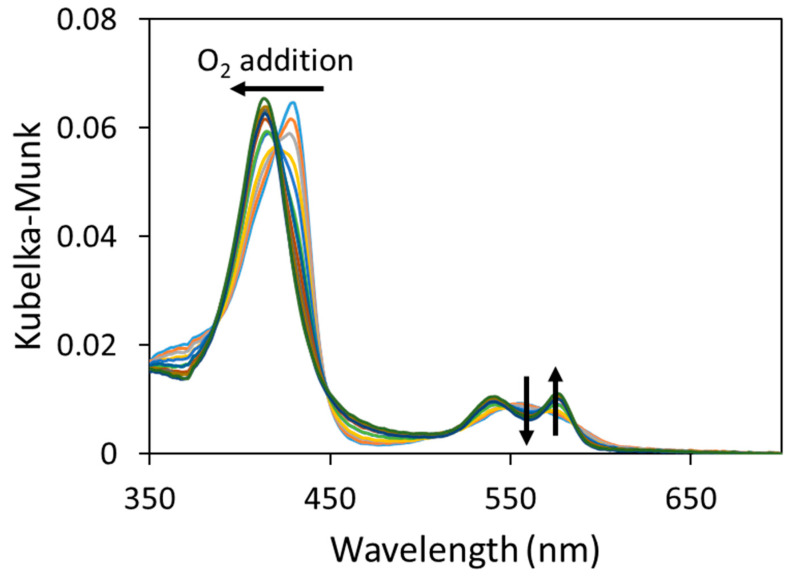
The Kubelka–Munk transformation of the UV–visible spectra of hemoglobin adsorbed on the polypropylene filter obtained using diffuse reflection. Oxygen additions and spectrum measurements were repeated until the fully oxygenated form was reached. Arrows are oriented from the deoxyhemoglobin spectrum to the oxyhemoglobin spectrum.

In tonometry experiments, to measure the UV–visible spectra of Hb adsorbed on PP-filters, we used an integration sphere, we measured the diffuse reflection spectra (Figure S10), and we applied the Kubelka–Munk transformation [84,85] to obtain spectra proportional to absorption spectra (see Section 3.5). The oxygenated and deoxygenated Hb spectra were acquired and then partially oxygenated Hb spectra after successive additions of oxygen following the same protocol as that of Hb solutions (Figure 4). The presence of isobestic points highlighted the equilibrium of only deoxygenated/oxygenated forms of Hb adsorbed on the PP-filter and a preservation of the oxidation state of its heme-iron. Due to the low intensity of the heme region, we only used the Soret band of these spectra to calculate the proportion of oxygenated hemoglobin.

**Figure 5:**
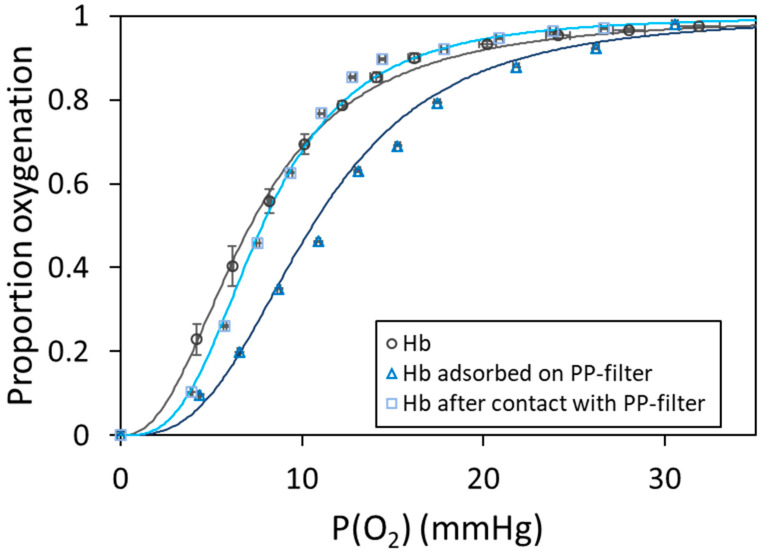
Oxygen binding curves: native hemoglobin (gray circles), hemoglobin adsorbed on the polypropylene filter (PP-filter) (light blue squares), and hemoglobin after contact with the PP-filter (dark blue triangles) in 0.1 mol.L^−1^ phosphate buffer pH 7.4. The fit using the Hill equation is shown (solid lines).

The oxygenation curve of Hb adsorbed on PP-filters is shown in Figure 5. Moreover, the oxygenation curve of native Hb in 0.1 mol.L^−1^ phosphate buffer pH 7.4 measured using the classical method of Hb absorption measurements is also provided for comparison. The data points used for native Hb were averaged over five experiments conducted under the same conditions. The sigmoidal shape of the curves reflects the cooperative binding of oxygen by the hemoglobin tetramer, and the difference between the two Hb oxygenation curves reflects the effect of Hb adsorption on the PP-filters. This effect can be quantified by the oxygen partial pressure at half saturation (P_50_) and the Hill coefficient determined by fitting the experimental curves with the Hill equation (see Equation (4)). The P_50_ and n values are given in Table 4.

The affinity of Hb adsorbed on the PP-filter decreased significantly, as shown by the increase in P_50_ from 7.09 ± 0.48 to 10.57 ± 0.42 mm Hg. Adsorbed Hb still exhibited cooperative oxygen binding, as confirmed by the Hill coefficients of 2.46 ± 0.02 and 2.96 ± 0.10. We have extensively discussed the consequences of protein adsorption on MNPs (see Section 2.2.2) [50,51,52,53,60,61,62,78,79,80,81,82,83]. Multispectroscopic investigations of interactions between Hb and PEMPs or polystyrene NPs have reported Hb conformational modifications after Hb adsorption. Specifically, circular dichroism has shown changes of the secondary structure of Hb after adsorption [60,61,62]. Our results suggest that the decrease in Hb affinity that we measured on adsorbed Hb on PP-filters comes from structural and conformational alterations after Hb adsorption on microstructured plastics. The cooperative behavior of adsorbed Hb on PP-filters removes the possibility of tetramer dissociation during adsorption on PP-filters. Interestingly, the structural analysis of porcine oxyHb adsorption on silica nanoparticles revealed a significant loss of secondary structure [65]. However, despite structural changes, adsorbed Hb on SiO_2_ nanoparticles exhibits higher oxygen affinity and lower cooperativity. As mentioned above, the relationship between the structural and functional modifications of adsorbed proteins is complex and depends on the surface chemistry of nanomaterials; protein activity can decrease or increase [65].

In this study, we measured the affinity decrease in Hb adsorbed on PP-filters, but we could not quantify the Hb oxygenation on aged microstructured plastics. Interestingly, a recent article showed that microplastic aging could affect their chemical and biological behavior, and aged PE microplastics were more cytogenotoxic and oxidative than virgin ones [86].

The oxygenation curve of Hb after contact with PP-filters is also shown in Figure 5. Surprisingly, the curve does not match that of native Hb. The sigmoidal shape reveals the cooperative oxygen binding and a decrease in Hb affinity for oxygen. However, although the Hill coefficient (3.01 ± 0.05) confirms the first observation, the P_50_ value is not significantly different from that of native Hb, 7.81 ± 0.46 for 7.09 ± 0.48 mmHg (Table 4). There are several hypotheses to explain a potential slight affinity decrease in Hb for oxygen after contact with PP-filters. Interactions without adsorption but after contact between oxyHb and PP-filters may cause irreversible structural and activity changes, or adsorbed oxyHb on PP filters may be desorbed due to weak and/or few interaction forces between protein and microstructured plastics. Thus, the non-adsorbed Hb solution may be a mixture of always-free oxyHb molecules and desorbed oxyHb molecules having irreversible structural modifications due to the adsorption on PP-filters. In contrast, the structural and functional modifications of porcine oxyHb are fully reversible after desorption from silica nanoparticles [65].

**Figure 6:**
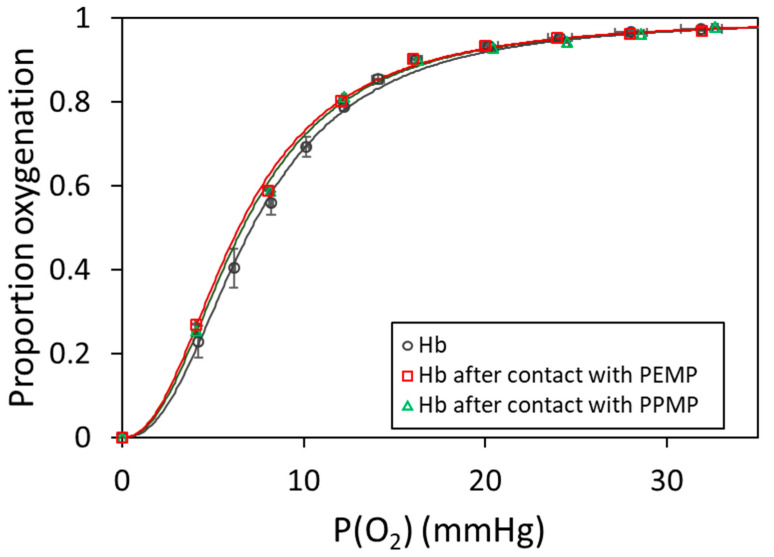
Oxygen binding curves: native hemoglobin (Hb) (gray circles), Hb after contact with polyethylene microparticles (PEMPs) (red squares), and Hb after contact with polypropylene microparticles (PPMPs) (green triangles) in 0.1 mol.L^−1^ phosphate buffer pH 7.4. The data were fit to the Hill equation (solid lines).

The oxygenation curves of hemoglobin after contact with PEMPs and Hb after contact with PPMPs are shown Figure 6. The curves are not very different from that of native Hb. The sigmoidal shapes reveal the cooperative binding and a slight increase in Hb affinity for oxygen. The Hill coefficient was 2.24 ± 0.03 for Hb after contact with PEMPs and 2.28 ± 0.03 for Hb after contact with PPMPs, but the P_50_, respectively, 6.43 ± 0.46 and 6.64 ± 0.46 mmHg were not significantly different from that of native Hb 7.09 ± 0.48 (Table 4).

To explain a possible slight affinity increase in Hb for oxygen after contact with PEMPs and PPMPs, there are two hypotheses: (1) Hb interacts with microparticles without adsorption or (2) reversible Hb adsorption on microparticles causes irreversible structural changes. However, these modifications after contact with PEMPs and PPMPs are certainly different from those after contact with PP-filters because Hb affinity for oxygen increased after contact with PEMPs and PPMPs but decreased after contact with PP-filters. These results showcase once again the complexity of the relationship between the structural and functional variations after adsorption on micro- and nanomaterials.

## 3. Materials and Methods

### 3.1. Virgin and Aged Microstructured Plastics

We used virgin microplastics from the same batch, as in our previous study [57]. Polyethylene microparticles (PEMPs) and polypropylene microparticles (PPMPs) were Ceridust 3610 and Ceridust 6050M (Clariant, Frankfurt, Germany) plastics. The polypropylene filters (Puradisc 25 mm) were purchased from Whatman (Springfield Mill, UK) with a pore size of 0.45 μm. Because these filters were sold in polypropylene frames, the edges were cut to excise the porous membrane. For the experiments, 10 × 15 mm^2^ pieces (PP-filters) were prepared and used.

The aging of plastics happens through impurities present in their structures, which can initiate their oxidation. Once the plastic is oxidized by oxygen present in the air, a CO radical is formed and, depending on the reaction pathway, can result in the production of different functional groups [66,67]. The methods we used to age virgin plastic microparticles oxidized around 4% of the carbon atoms in the particles, which is consistent with reported degrees of oxidation of plastics under natural alteration [87]. After testing several aging methods (aging in sea water with mechanical stress and illumination at 4 °C, heating, gamma, and electron irradiation), we chose heating for PPMPs and electron irradiation for PEMPs. Electron irradiation was the only method that aged PEMPs effectively, but it started melting PPMPs. Heating was efficient to age PPMPs. To age PPMPs, a 100 mL beaker was filled with microparticles and left in an oven at 85 °C for two months. To age PEMPs, microparticles were irradiated using 10 MeV electrons provided from ALIENOR facility in Saclay (NIMBE Laboratory, Gif-Sur-Yvette, France). PEMPs were placed in a quartz SUPRASIL cuvette (10 mm by 10 mm) and irradiated with 12 ns pulses (20 Gy per pulse) at a frequency of 10 Hz. PEMPs were irradiated four times with 500 kGy doses and were mixed between each irradiation to allow for uniform aging of the sample.

### 3.2. Characterization of Microstructured Plastics

Virgin plastic microparticles (PEMPs and PPMPs) were extensively characterized using optical microscopy, small-angle X-ray scattering (SAXS), X-ray photoelectron spectroscopy (XPS), Fourier-transform infrared spectroscopy (FTIR), and Raman confocal microscopy in ref. [57], which provides details on the characterization methods. Aged plastic microparticles (ag-PEMPs and ag-PPMPs) were similarly characterized using the same techniques and methods.

PP-filters were characterized using SAXS, XPS, FTIR, and Raman microscopy. The reported SAXS data were an average of two measurements under vacuum carried out using a Xeuss 2.0 copper setup from XenocsTM with a counting time of 3600 s and a sample-to-detector distance of 2.5 m. XPS measurements on PP-filters were carried out using a Kratos Axis Ultra DLD spectrometer (Manchester, UK) with a monochromatic Al K_α_ excitation (1486.7 eV) and a charge compensation system. Survey spectra were acquired at analyzer pass energy of 160 eV and high-resolution spectra at pass energy of 40 eV. The binding energy scale was calibrated to C 1s line at 284.8 eV. The percentages of atomic concentration were calculated using CasaXPS version 2.3.19PR1.0. Peak fitting was performed after subtracting a Shirley background, and peak areas were corrected by taking into account the Scofield sensitivity factors. FTIR spectra of PP-filters were obtained using a Bruker Tensor 27 IR (Ettlingen, Germany) with a Specac Golden Gate ATR accessory. 128 scans were acquired from 500 to 5000 cm^−1^ with a resolution of 1 cm^−1^ and a scanning rate of 10 kHz. The Raman spectrum of PP-filters was acquired with a WITec alpha300 RA instrument (Oxford Instruments, Wiesbaden, Germany). A piece of filter was deposited on CaF_2_ substrate (ESCO Optics, Oak Ridge, NJ, USA). The spectrum was acquired 532 nm excitation, 100× objective (NA 0.9) 600 g.mm^−1^ grating, 5 mW laser power, an exposure time of 5 s, and 10 accumulations. Ten spectra were measured by moving 4 μm in the same direction between each measurement and averaged.

### 3.3. Hemoglobin Preparation

All solutions were prepared using pure Milli-Q water (18 MΩ.cm, TOC = 5 ppb, MilliPore, Burlington, MA, USA). The phosphate buffer was obtained by dissolving NaH_2_PO_4_ (>99%, 231-449-2, VWR Chemicals, Leuven, Belgium) and Na_2_HPO_4_ (>99%, 231-448-7, VWR Chemicals). The phosphate buffer used was 0.1 mol.L^−1^ pH 7.0 for adsorption experiments (maximum hemoglobin adsorption) and pH 7.4 for oxygenation experiments (physiological pH) [65]. The ACD (acid citrate dextrose) anticoagulant solution [88] was prepared by mixing 4.8 g.L^−1^ citric acid (>99.5%, 27487, Sigma, St Louis, MO, USA), 13.2 g.L^−1^ sodium citrate (>99%, S1804, Sigma), and 14.7 g.L^−1^ glucose (>99.5%, G7528, Sigma). A solution of sodium chloride (>99%, S9625, Sigma Aldrich, St Louis, MO, USA) was used at 9 g.L^−1^.

Hemoglobin (Hb) was prepared from pig (Sus scrofa domesticus) blood supplied by the Harang slaughterhouse (Houdan, France). The anticoagulant solution was mixed on site with the fresh blood (1:3 solution/blood (*v*/*v*)). Porcine Hb was purified at 4 °C in its oxygenated form, following modified standard process [89]. The blood was centrifuged (5000× *g*, 10 min) to remove the supernatant containing lipids via vacuum aspiration, and the NaCl solution was used to resuspend the red blood cells. This step was repeated three times. The red blood cells were hemolyzed via osmotic shock by adding (1:2) Milli-Q water. An amount of 2.8 M phosphate buffer pH 6.8 was added (1:9) to precipitate the red blood cell membranes. The solution was centrifuged (25,000× *g*, 20 min), and the supernatant containing oxyhemoglobin (oxyHb) was dialyzed extensively against Milli-Q water (3.5 kDa cut-off, Spectra/Por^®^3, Spectrum Labs, San Francisco, CA, USA). The oxyHb desalted solution was centrifuged (16,000× *g*, 5 min) and passed through a mixed-bed ion exchange AG 501-X8 resin (Bio-Rad, Hercules, CA, USA) to remove the 2,3-diphosphoglycerate (DPG), which is a natural effector of mammalian Hb [90]. After centrifugation (16,000× *g*, 5 min), the oxyHb concentration was determined on a Shimadzu UV-2600 spectrophotometer (Shimadzu, Kyoto, Japan) by measuring the absorption at 576 nm (ε = 15,150 M^−1^.cm^−1^) [91]. The purified oxyHb solution was kept in filled and sealed tubes to avoid the presence of oxygen and the oxyHb-iron oxidation. Solutions were stored at 4 °C for up to two weeks and centrifuged (16,000× *g*, 5 min, 4 °C) before use. The absence of methemoglobin was systematically verified by measuring the absorbance value ratio at 576 and 541 nm.

### 3.4. Adsorption Isotherms

Contact was carried out in 15 mL tubes containing 10 mL of an oxyHb solution in 0.1 mol.L^−1^ phosphate buffer pH 7.0 and 0.1 g of plastic microparticles (PEMPs, PPMPs, ag-PEMPs, ag-PPMPs). In aqueous solutions, the microplastics remained at the surface, but their dispersion was observed in presence of the protein, showing that Hb was adsorbed. The plastic microparticle concentration was kept the same (20 g.L^−1^), but the Hb concentrations were varied (from 5 mg.L^−1^ to 100 mg.L^−1^ for virgin microplastics and from 50 mg.L^−1^ to 1 g.L^−1^ for aged microplastics) to plot the isotherms. Because the protein stabilizes the dispersion of the microplastics, the exposure time was determined when the Hb concentration in solution was stable after its decrease due to adsorption on microparticles. The samples were kept on rotating wheels (3 rotations per minute) in a cold room (4 °C) for 72 h. The samples were centrifuged (3000× *g*, 10 min) to remove the plastic microparticles (the density of PEMPs is 0.97 and that of PPMPs is 0.90). The Hb amount left in solution was measured using absorption spectroscopy after contact with virgin microplastics and diffuse reflection spectroscopy (see Section 3.5) after contact with aged microplastics. Equilibrium adsorption isotherms were obtained using the depletion method. The amount of adsorbed Hb was deduced from the initial amount of Hb and the amount of non-adsorbed Hb. This process was repeated a total of three times, and the results were averaged.

The isotherms were fitted using the Langmuir model (Equation (2)):

(2) $$\begin{array}{c} m_{ads}=\frac{m_{\infty }\;\times \;K_{ads}\;\times \;C}{1+K_{ads}\;\times \;C},\; \end{array}$$

where $m_{ads}$ (mg.m^−2^) is the amount of adsorbed protein per unit of specific surface of plastic microparticles, C is the concentration of non-adsorbed protein, $m_{\infty }$ (mg.m^−2^) is the maximum amount of adsorbed protein, and $K_{ads}$ (L.g^−1^) is the adsorption constant, a higher value corresponding to a higher affinity between protein and plastic. In our case, *m*_∞_ and $K_{ads}$ are the fit parameters, and the values we obtained can be compared with values from the literature. For the aged microplastics, only the data points corresponding to the increase in adsorbed Hb were considered for the fit.

Hb adsorption isotherms were only measured with microparticles because the specific surface area of the PP-filters is too small to reach a significant loss of the non-adsorbed Hb amount. In other words, considering the specific surface area of PP-filters, obtaining the same available surface area as with PPMPs would have required 10 × 90 mm^2^ PP-filters.

### 3.5. Hemoglobin Oxygenation

In the case of Hb oxygenation in solution, 3 mL of oxyHb (1 g.L^−1^) in 0.1 mol.L^−1^ phosphate buffer pH 7.4 was introduced in a glass tonometer (80 mL). The Hb visible spectrum was measured before and after leaving the Hb under argon flow (>99.9999%, BIP^®^Ar, Air Products, Allentown, PA, USA) for 2 h to remove the oxygen. Once oxyHb was deoxygenated, a controlled volume of oxygen was added using a gas-tight syringe (Hamilton, Franklin, MA, USA), and after five minutes, the visible spectrum was measured. The oxygen addition and spectrum measurement were repeated until the solution was almost completely reoxygenated. The tonometer was left open for 10 min to complete the reoxygenation, and the visible spectrum was measured again.

**Figure 7:**
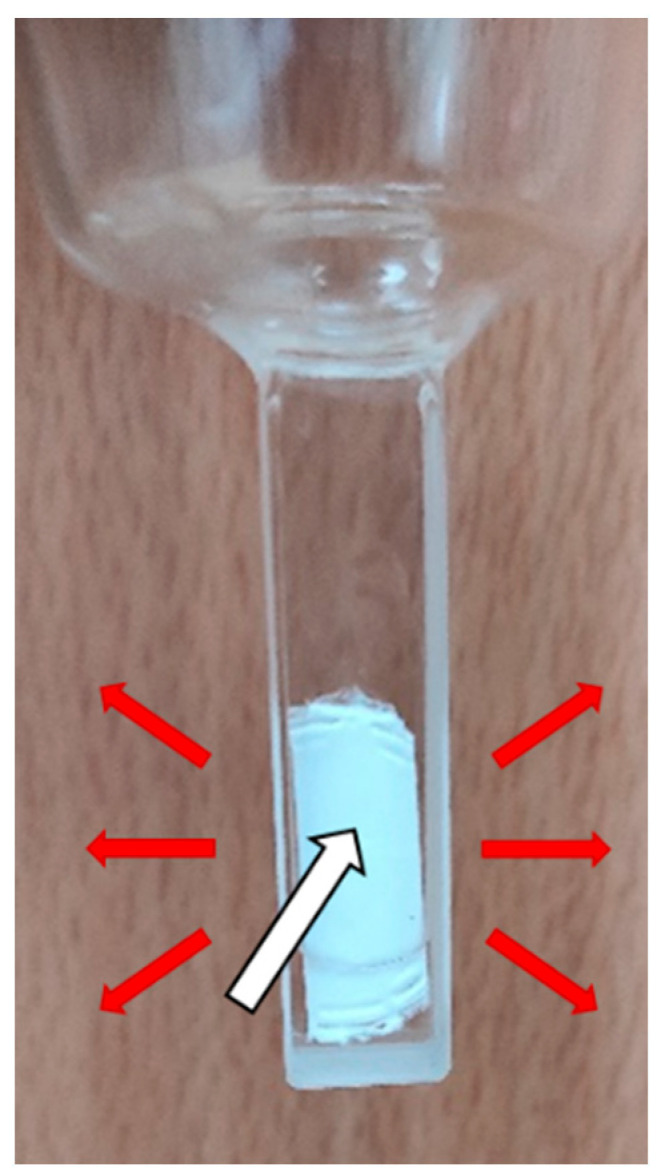
A polypropylene filter in a tonometer with 100 µL of 0.1 mol.L^−1^ phosphate buffer. The red color of adsorbed hemoglobin can barely be seen with the naked eye, but it can be detected and analyzed through diffuse reflection symbolized by the red arrows. The white arrow represents incident light.

For the oxygenation of adsorbed Hb on PP-filters, we used a piece of filter (10 × 15 mm^2^) that had been attached to the top of a 15 mL tube containing 10 mL of oxyHb solution (20 g.L^−1^) in 0.1 mol.L^−1^ phosphate buffer pH 7.4. The tube was left on rotating wheel (3 rotations.min^−1^) in a cold room (4 °C) for 72 h. After exposure, the PP-filter was removed from the tube and patted with absorbent paper to remove the solution left on the surface and keep only the adsorbed Hb. The PP-filter was introduced in a tonometer with 100 μL of 0.1 mol.L^−1^ phosphate buffer pH 7.4 to prevent Hb from drying out (Figure 7). Oxygenation measurements were conducted with the same method as for Hb in solution but using an ISR-2600PLUS integration sphere mounted on a UV-2600 spectrophotometer to acquire the spectra. These spectra correspond to the diffuse reflection of light, so the Kubelka–Munk transform [84,85] was applied to obtain a signal proportional to Hb absorption. To the best of our knowledge, this is the first time that diffuse reflection spectroscopy has been to measure Hb oxygenation curves.

In both cases, i.e., Hb oxygenation in solution and oxygenation of adsorbed Hb on PP-filters (at 22 °C), using the spectra measured after Hb deoxygenation and after complete Hb reoxygenation as references, we calculated the proportion of oxyHb in a sample using its spectrum (Equation (3)):

(3) $$\begin{array}{c} \theta =\frac{A\left(\lambda \right)-A_{ox}\left(\lambda \right)}{A_{deox}\left(\lambda \right)-A_{ox}\left(\lambda \right)}, \end{array}$$

where θ is the proportion of oxyHb in the sample, $A\left(\lambda \right)$ is the absorbance value of the sample at a given wavelength, and $A_{ox}\left(\lambda \right)$ and $A_{deox}\left(\lambda \right)$ are the absorbance values of the solution of fully oxygenated Hb and fully deoxygenated Hb, respectively. We used this formula for all wavelengths that correspond to the Hb signal, excluding isobestic points, and then averaged the results to obtain a more accurate value.

We calculated the values of $\log\left(\frac{\theta }{1-\theta }\right)$ depending on $\log\left(P_{O_{2}}\right)$ and used the linearized form of the Hill equation (Equation (4)):

(4) $$\begin{array}{c} \log\left(\frac{\theta }{1-\theta }\right)=n\times \log\left(P_{O_{2}}\right)-n\times \log\left(P_{50}\right), \end{array}$$

where θ is the proportion of oxyHb in the sample, $P_{O_{2}}$ is the partial pressure of O_2_ in the system, n is the Hill coefficient, and P_50_ is the partial pressure of O_2_ at which half the Hb is oxygenated. We performed a linear regression using Excel 2016 version 16.0.5448.1000 (32 bits) to calculate *n* and P_50_. For the control samples, we averaged the data on five samples before calculating *n* and P_50_.

PEMPs and PPMPs were not used for tonometry. Due to the low density of plastic particles, particles quickly rise (cream) during oxygenation experiments, which prevents measurements in solution [57].

## 4. Conclusions

We investigated porcine hemoglobin adsorption on microstructured plastics, and we analyzed the functional modifications of the adsorbed protein. Hb adsorption was measured on model virgin and aged PE and PP microplastics, and Hb oxygenation was carried out on a polypropylene filter using a novel method involving the diffuse reflection of the light. Indeed, the creaming phenomenon of microparticles precluded using the classical method of Hb oxygenation in solution, and the low amounts of adsorbed Hb on filters make adsorption measurements impossible. The PEMPs were aged with 2 MGy electron irradiation and the PPMPs with a heat treatment at 85 °C for 2 months.

The microscopy and SAXS analysis of the microstructured plastics made it possible to determine their median diameter and specific surface. ATR-FTIR and XPS investigations highlighted that the plastics did not contain additives, but most of them were partially oxidized. Virgin PEMPs contained 0.9% oxygen atoms compared to the amount of carbon atoms where both aged PEMPs and PPMPs contained 2.6% oxygen atoms on their surface. Moreover, aging caused an increase in the diameter of PEMPs and a homogenization of PPMPs but led to no significant changes in the specific surface area of microparticles.

In addition, our study demonstrates that aged plastics can have different biological effects from virgin plastics. Although the characteristics of aged microparticles are not very different from those of virgin microparticles, there are nevertheless notable differences. In particular, aged microparticles are partially oxidized and contain a significant amount of oxygen atoms. Interactions with biological materials obviously depend on the surface chemistry of plastics, and to better reflect the reality of plastics in the environment, we recommend including aged MNPs in studies. Our measurements of adsorption isotherms undoubtedly illustrate this last point. Compared with virgin PEMPs and PPMPs, after the contact of aged microparticles with hemoglobin, the cream was less stable, and the microparticles returned more easily into solution. Therefore, Hb has a lower affinity for aged microplastics but can be adsorbed in higher quantities on aged plastics. Thus, protein adsorption is controlled in part by the aging of plastics. In comparison with BSA adsorption [57], the protein charge may also control the adsorption on plastics.

The oxygenation curves of adsorbed hemoglobin on PP-filters kept their typical sigmoidal shape, suggesting that cooperativity, the Hb tetramer, and the heme oxidation state are all maintained. However, the activity of adsorbed Hb on PP-filters decreased. This result is consistent with the structural analysis of adsorbed Hb from the literature, showing changes of the secondary structure and conformational modifications of Hb. Similarly to silica nanoparticles, MNPs can be considered to be a new stress and act as an alternative effector for Hb [65]. Our results also suggest that Hb adsorption is reversible, but structural modifications may be irreversible.

## Figures and Tables

**Table 1:** Characteristics of virgin microstructured plastics used for experiments: polyethylene microparticles (PEMPs), polypropylene microparticles (PPMPs), and PP-filters. *FTIR spectra of PEMPs, PPMPs, and PP-filters indicate that there are no additives or impurities. d, median Feret diameter; S, specific surface area; CI, carbonyl index.

Characterization Method	PEMP [57]	PPMP [57]	PP-Filter
Optical microscopy	d = 4.2 µm	d = 10.0 µm	
SAXS	S = 2.5 m^2^.g^−1^	S = 1.1 m^2^.g^−1^	S = 0.7 m^2^.g^−1^
ATR-FTIR*	CI = 0.00–0.02	CI = 0.02 ± 0.02	CI = 0.09 ± 0.02
XPS	0.9% oxygen	No oxygen atoms	No oxygen atoms
Raman microscopy	3 crystalline phases	1 crystalline phase	1 crystalline phase

**Table 2:** Characteristics of aged microstructured plastics used for experiments: aged polyethylene microparticles (ag-PEMPs) and aged polypropylene microparticles (ag-PPMPs). d, median Feret diameter; S, specific surface area; CI, carbonyl index.

Characterization Method	ag-PEMP	ag-PPMP
Optical microscopy	d = 5.8 µm	d = 9.9 µm
SAXS	S = 2.4 m^2^.g^−1^	S = 1.2 m^2^.g^−1^
ATR-FTIR	CI = 0.73 ± 0.04	CI = 1.15 ± 0.04
XPS	2.6% oxygen atoms	2.6% oxygen atoms
Raman microscopy	3 crystalline phases	1 crystalline phase

**Table 3:** The parameters of oxyhemoglobin adsorption on virgin polyethylene (PE) and polypropylene (PP) microparticles (PEMPs and PPMPs, respectively) and aged PE and PP microparticles (ag-PEMPs and ag-PPMPs, respectively) calculated by fitting the adsorption isotherms shown in Figure 2 and in Figure 3 to the Langmuir model: the maximum amount of adsorbed hemoglobin (m_∞_) and the adsorption constant (K_ads_).

	PEMP	ag-PEMP	PPMP	ag-PPMP
m_∞_ (mg.m^−2^)	1.0 ± 0.1	2.2 ± 0.2	2.2 ± 0.2	4.1 ± 0.2
K_ads_ (L.mg^−1^)	26 ± 3	0.021 ± 0.005	92 ± 8	0.014 ± 0.002

**Table 4:** The oxygen partial pressure at half saturation (P_50_) and the Hill coefficient (n) of hemoglobin (Hb) oxygen binding for the studied samples, from top to bottom: native Hb, Hb adsorbed on polypropylene filter (PP-filter), Hb after contact with the PP-filter, Hb after contact with polyethylene microparticles (PEMPs), hemoglobin after contact with polypropylene microparticles (PPMPs).

Sample	P_50_ (mmHg)	n
Hb	7.09 ± 0.48	2.46 ± 0.02
Hb on PP-filter	10.57 ± 0.42	2.96 ± 0.10
Hb after PP-filter	7.81 ± 0.46	3.01 ± 0.05
Hb after PEMP	6.43 ± 0.46	2.24 ± 0.03
Hb after PPMP	6.64 ± 0.46	2.28 ± 0.03

## Data Availability

The data can be obtained from the authors.

## Supplementary Materials

The following supporting information can be downloaded at https://www.mdpi.com/article/10.3390/ijms25137047/s1. Reference [92] is cited in the Supplementary Materials.
