# Supplementary material for: The Impact of Virgin and Aged Microstructured Plastics on Proteins: The Case of Hemoglobin Adsorption and Oxygenation
Source: Int J Mol Sci. 2024 Jun 27;25(13):7047. doi: 10.3390/ijms25137047 (PMC11241625; doi:10.3390/ijms25137047)
Supplement: Supplementary file 1 [file ijms-25-07047-s001.zip › ijms-3055960-SI.pdf]

Supporting Information

The Impact of Virgin and Aged Microstructured Plastics on Proteins: The Case of Hemoglobin Adsorption and Oxygenation

Florent Saudrais [1], Marion Schvartz [1], Jean-Philippe Renault [1], Jorge Vieira [1], Stéphanie Devineau [1,2], Jocelyne Leroy [1], Olivier Taché [1], Yves Boulard [3] and Serge Pin [1,*]

[1] NIMBE, CNRS, CEA, Université Paris-Saclay, 91191 Gif-sur-Yvette, France; florent.saudrais@cea.fr (F.S.)

[2] Unité de Biologie Fonctionnelle et Adaptative, CNRS, Université Paris Cité, 75013 Paris, France

[3] Institute for Integrative Biology of the Cell (I2BC), CNRS, CEA, Université Paris-Saclay, 91198 Gif-sur-Yvette, France; yves.boulard@cea.fr

* Correspondence: serge.pin@cea.fr

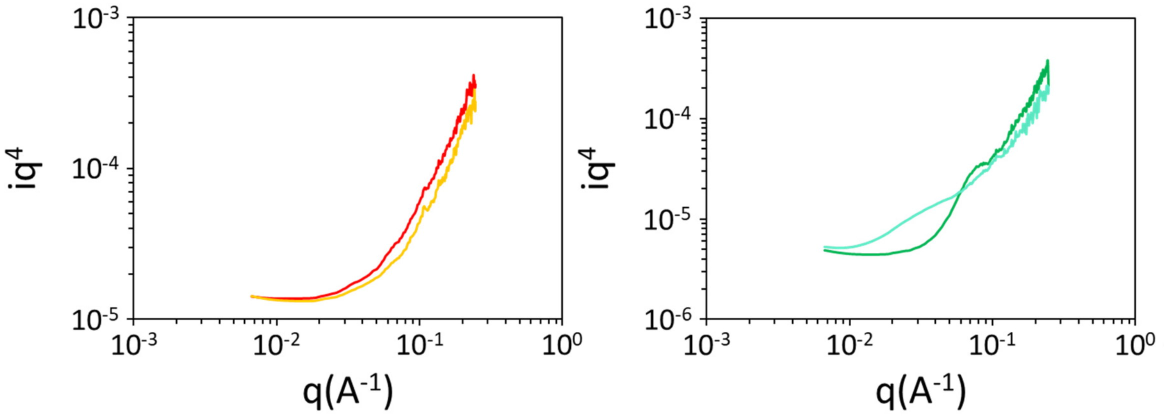

Figure S1: SAXS spectra of PEMPs (red) and aged PEMPs (orange) on the left, PPMPs (dark green) and aged PPMPs (teal) on the right.

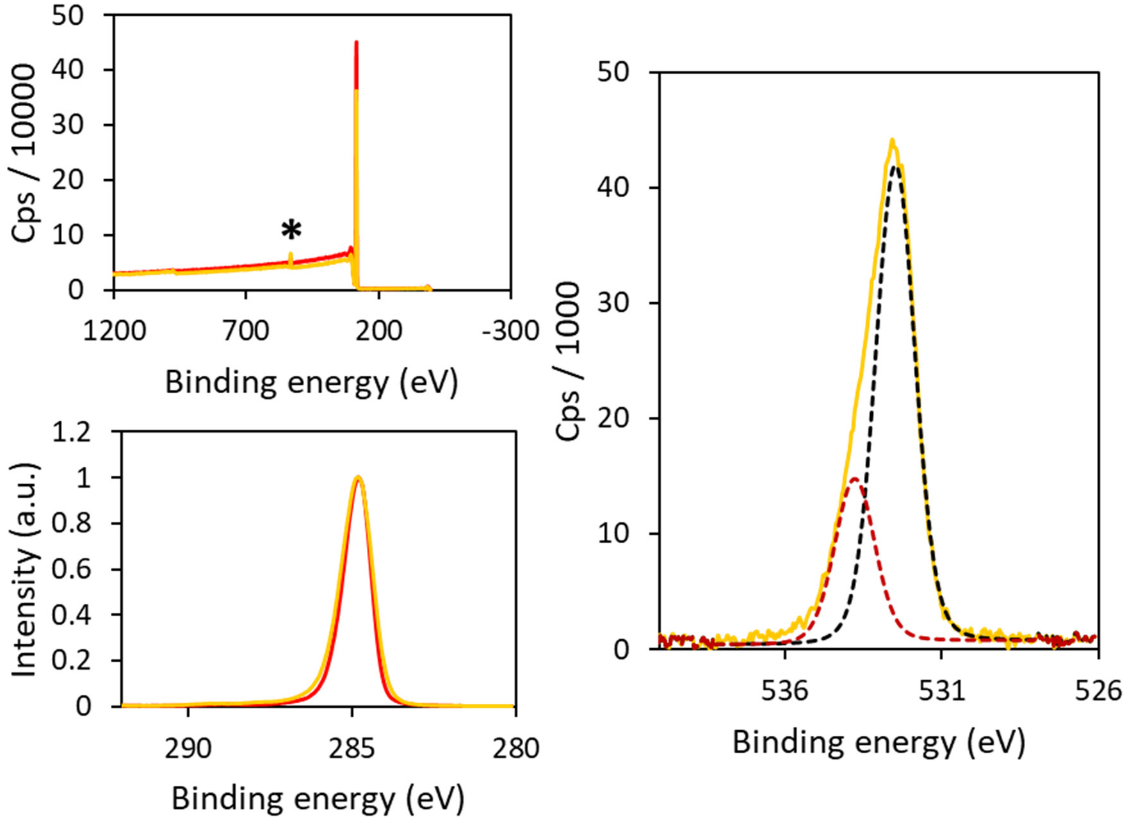

Figure S2: XPS spectra of PEMPs (red) and aged PEMPs (orange). The top-left figure corresponds to global spectra with the oxygen peak indicated by an asterisk, bottom-left figure shows the high-resolution carbon peaks normalized with a corrected baseline, and the right-hand figure shows the high-resolution oxygen peak with a corrected baseline and the components used for its fit (red and black).

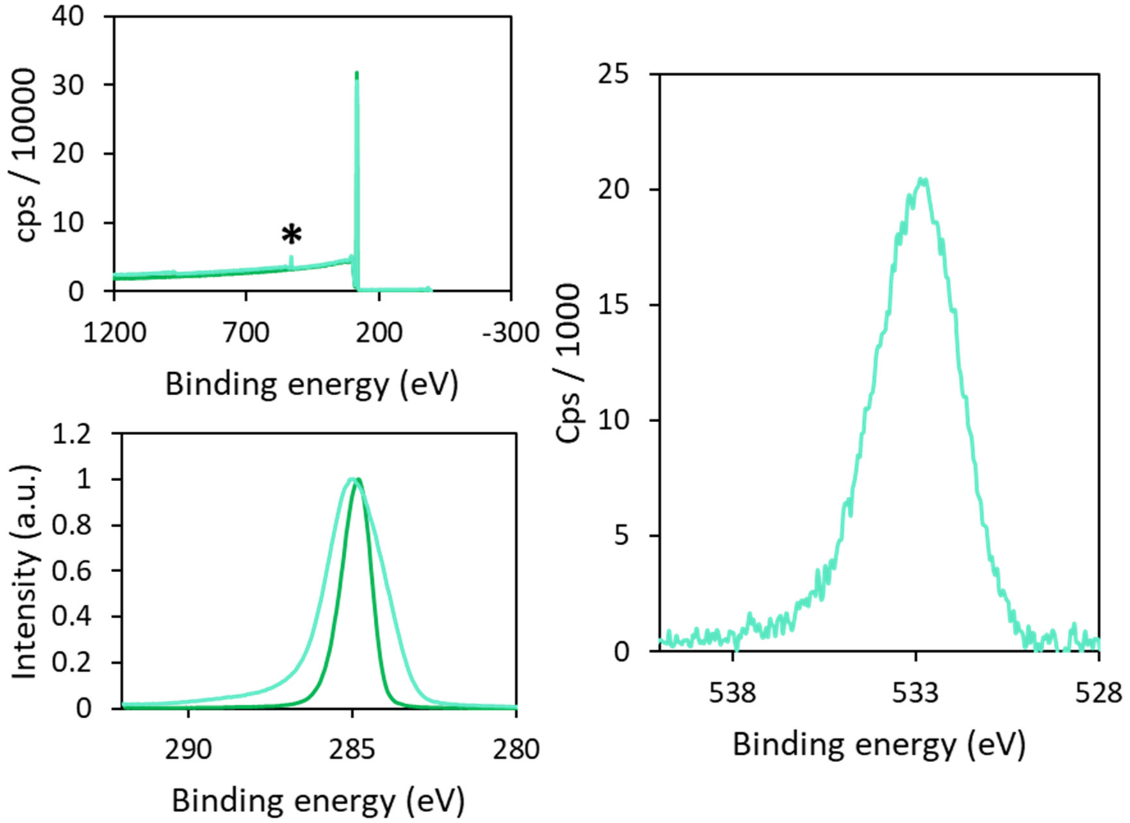

Figure S3: XPS spectra of PPMPs (dark green) and aged PPMPs (teal) The top-left figure corresponds to global spectra with the oxygen peak indicated by an asterisk, bottom-left figure gives the high-resolution carbon peaks normalized with a corrected baseline, and right-hand figure shows the high-resolution oxygen peak with a corrected baseline.

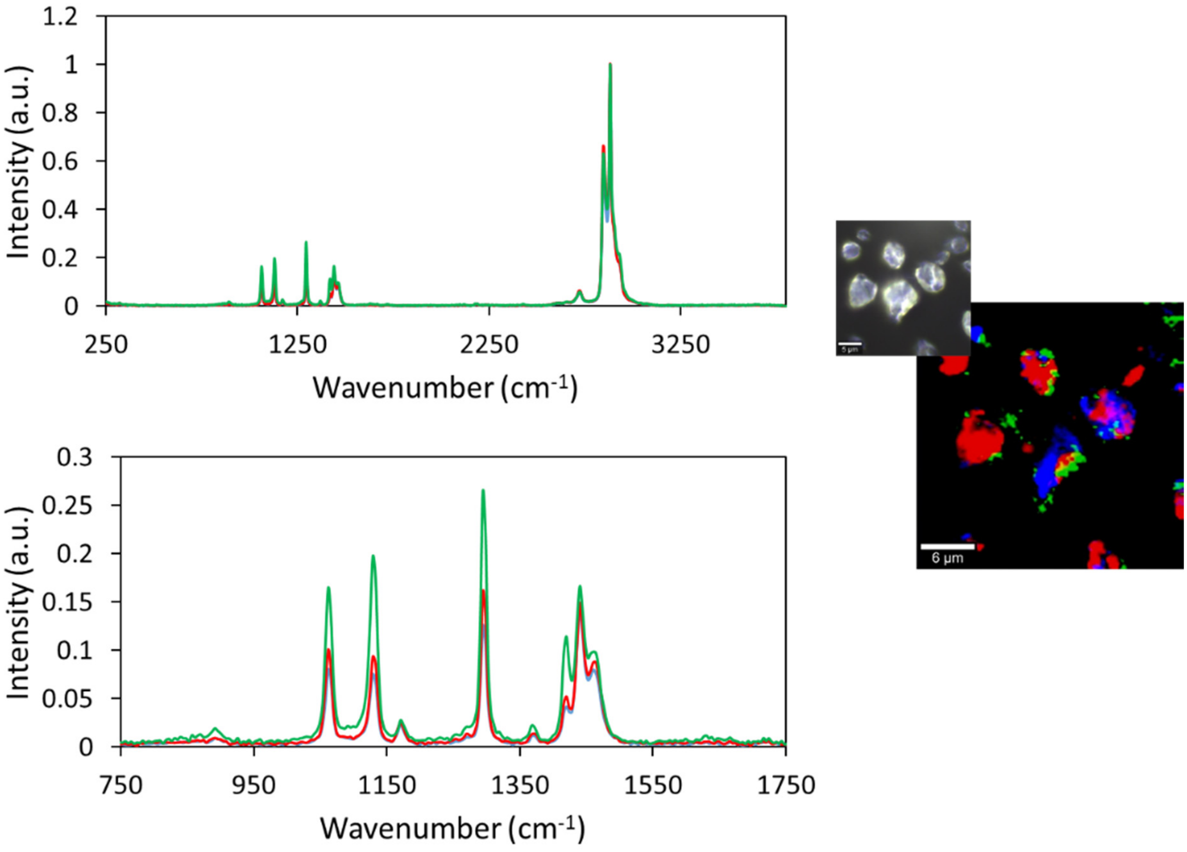

Figure S4: On the left, Raman spectra of the three components observed during Raman imaging experiments on aged PEMPs. Full spectrum on top and zoom on the Raman spectrum from 750 cm^{-1} to 1750 cm^{-1} on the bottom. On the right, result of Raman imaging experiments on aged PEMPs, each component being represented by the same color on the spectra and the image.

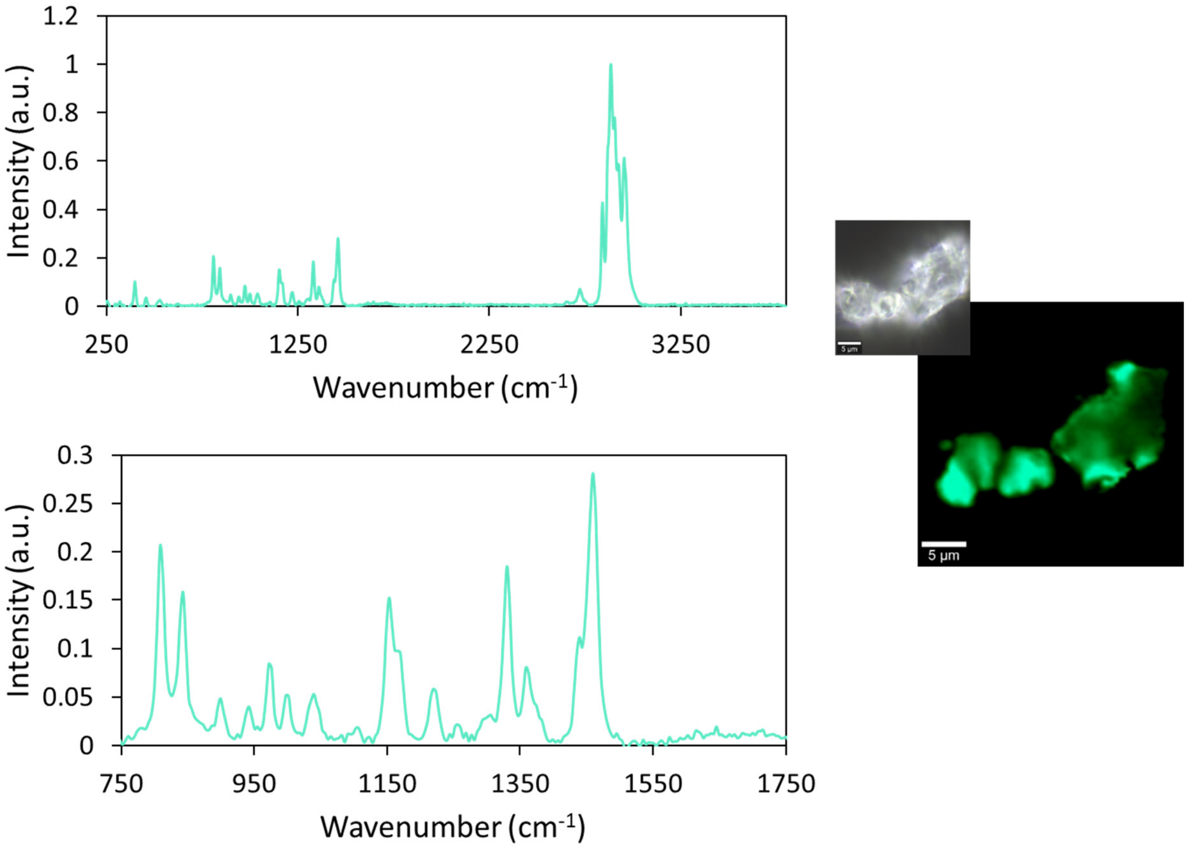

Figure S5: On the left, Raman spectra observed during Raman imaging experiments on aged PPMPs. Full spectrum in the upper figure and zoom on the Raman spectrum from 750 cm^{-1} to 1750 cm^{-1} in the lower figure. On the right, result of Raman imaging experiments on aged PPMPs.

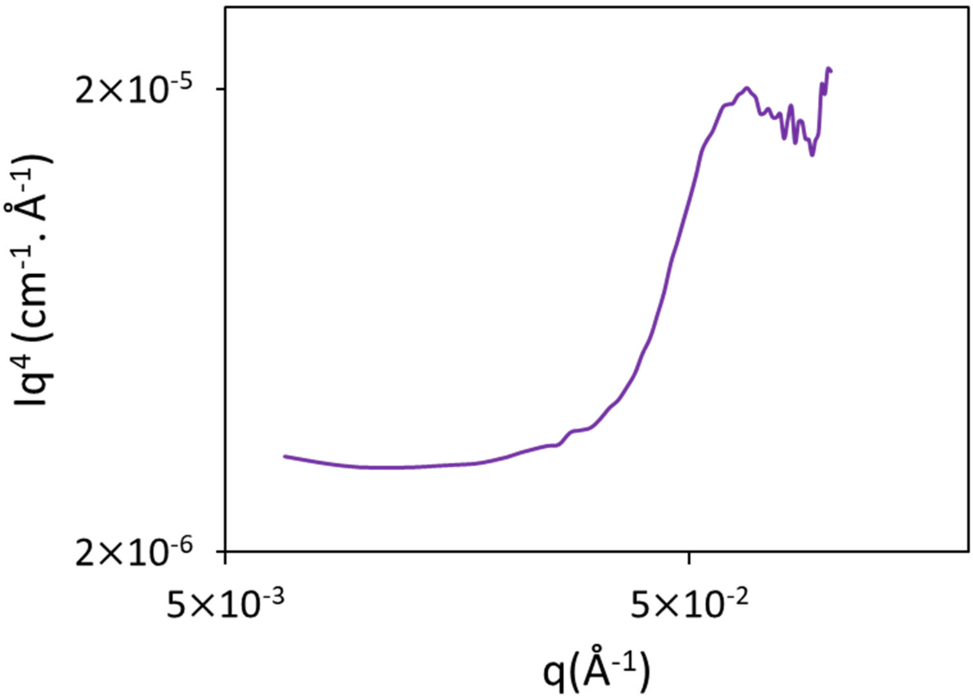

Figure S6: SAXS spectrum of a PP filter adapted from [92], measured in a vacuum 2.5 m away from the detector with 3600 seconds of accumulation.

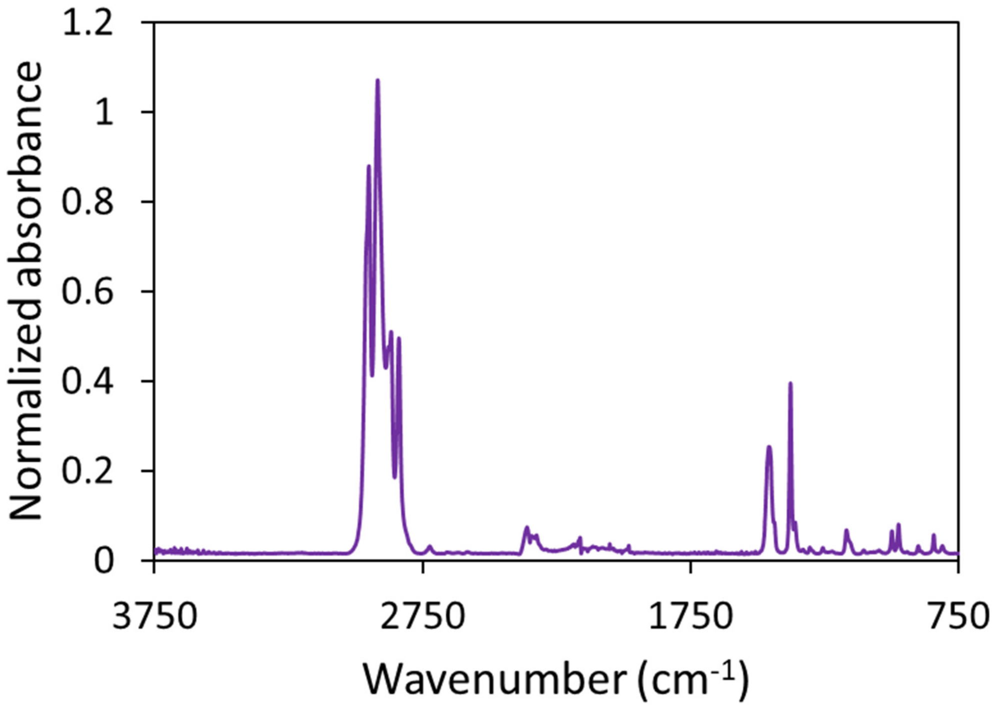

Figure S7: ATR-FTIR spectrum of a PP filter. The noise between 2000 cm^{-1} and 2500 cm^{-1} corresponds to the CO_2 signal.

Wavenumber (cm^{-1})	Attribution
840	γ $C–CH_3$
972	ρ $–CH_3$
997	ρ $–CH_3$
1165	ρ $–CH_3$
1375	δ_s $–CH_3$
1455	δ_s $–CH_2–$
2838	γ_s $–CH_2–$
2917	γ_a $–CH_2–$
2952	γ_a $–CH_3$

Table S1: Peak attributions of the ATR-FTIR spectrum of a PP filter.

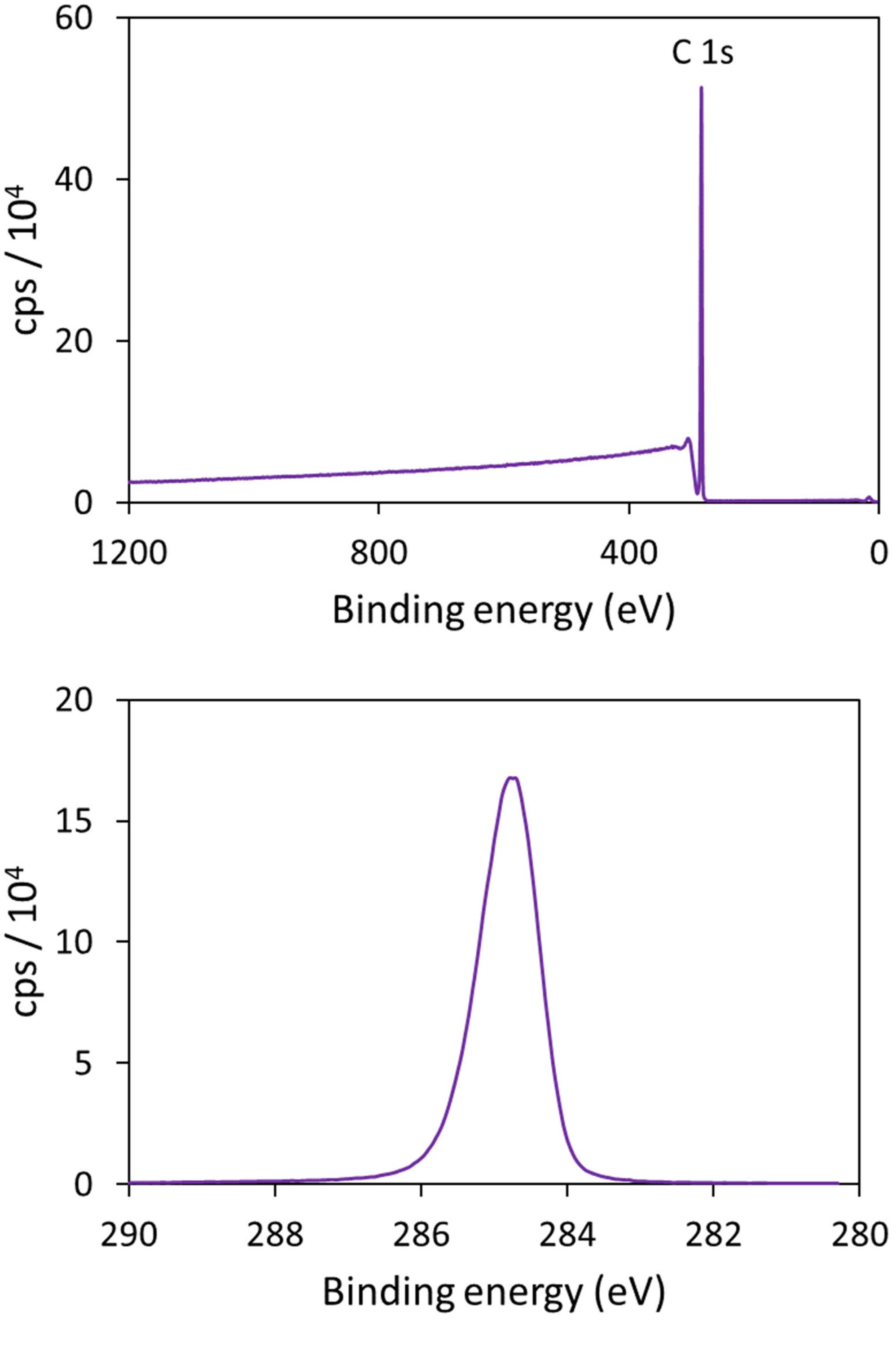

Figure S8: XPS spectrum of a PP filter and corresponding orbital (top), and high-resolution spectrum of the carbon peak with a corrected baseline (bottom).

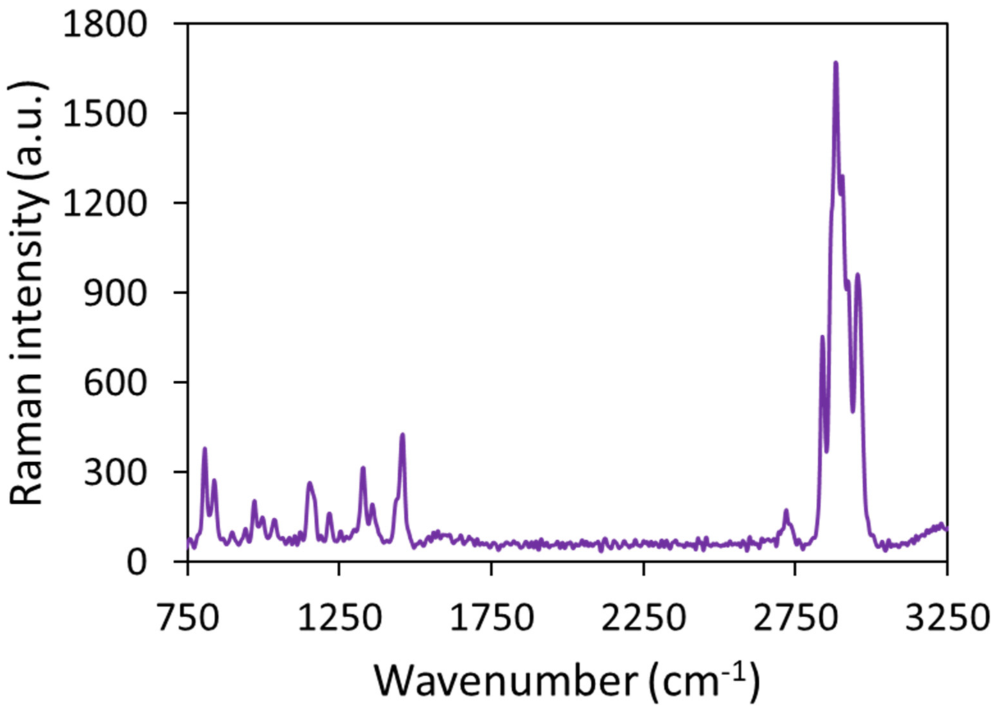

Figure S9: Raman spectrum of a PP filter.

Wavenumber (cm^{-1})	Attribution
398	Expansion carbon chain
809	γ C-C (crystalline phase)
840	γ C-C (amorphous phase)
976	ρ CH_3
1157	ρ CH_3
1218	γ C-C carbon chain
1328	δ CH / τ CH_2
1457	δ_a CH_3
2840	γ_s CH_2
2883	γ_s CH_2
2957	γ_s CH_2

Table S2: Peak attributions of the Raman spectrum of a PP filter.

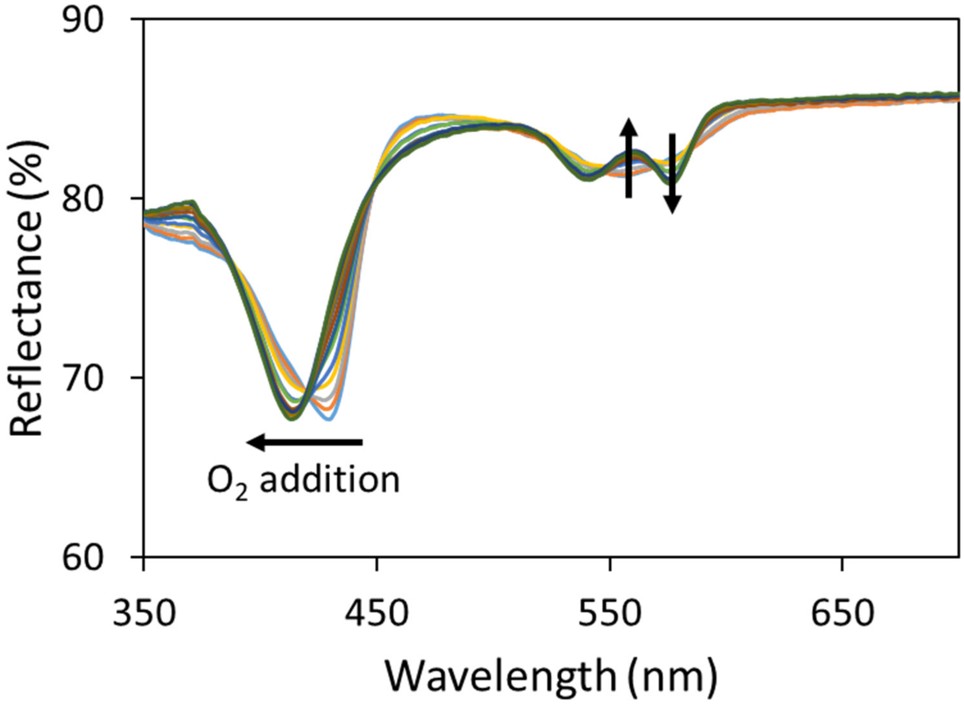

Figure S10: Visible spectra of hemoglobin adsorbed on a PP filter after each oxygen injection, measured using diffuse reflection.
